# Ketogenic Metabolic Therapies for Psychiatric and Neurodevelopmental Disorders

**DOI:** 10.1192/bjo.2025.10128

**Published:** 2025-06-20

**Authors:** Cristina Fante, Franziska Spritzler, Dorian Greenow, Sofia Deloudi

**Affiliations:** ^1^MetaboliCo GmbH, Bern, Switzerland; ^2^Keto-Mojo, Napa, USA; ^3^Metabolic Health Consulting GmbH, Zurich, Switzerland

## Abstract

**Aims:** Emerging evidence suggests that disruptions in brain energy metabolism – including impaired insulin signalling, altered glucose utilization, and mitochondrial dysfunction – may contribute to psychiatric and neurodevelopmental disorders such as bipolar disorder, schizophrenia, depression, post-traumatic stress disorder (PTSD), and autism spectrum disorder (ASD).

Ketogenic metabolic therapies (KMTs) provide the brain with an alternative energy source in the form of ketone bodies, which have been hypothesized to restore metabolic balance and improve psychiatric symptoms. Here we review the potential therapeutic effects of KD for mental health.

**Methods:** A structured review of recent clinical research was conducted to evaluate the influence of KMT on psychiatric and neurodevelopmental disorders. Relevant studies were identified through a manual selection process.

**Results:** Across studies, KMTs were associated with improvements in both psychiatric and metabolic outcomes. Patients with severe mental illness – like schizophrenia and bipolar disorder – demonstrated symptom reduction, decreased psychotropic medication use, and, in some cases, remission. Individuals with metabolic impairments experienced resolution of metabolic syndrome criteria alongside psychiatric symptom improvements. Case series also indicate that KMTs may support symptom remission in depression and anxiety. Early clinical research and preliminary findings indicate the feasibility and potential benefits of KMTs in PTSD and ASD. Most studies monitored adherence to KMT through ketone testing, recognizing adherence as a key factor in achieving therapeutic outcomes.

**Conclusion:** These findings highlight the potential of KMTs as adjunctive treatments in psychiatry. Symptom improvements across mood disorders, PTSD, and ASD, along with metabolic benefits, warrant further clinical investigation. Metabolic psychiatry presents a novel approach to understanding and treating these conditions by targeting brain energy metabolism.

